# Evaluating the Effect of Acceptance and Commitment Therapy on Post-traumatic Growth in Cancer Survivors: A Pilot Non-randomized Controlled Trial in a Greek Sample

**DOI:** 10.7759/cureus.102873

**Published:** 2026-02-03

**Authors:** Mara Gkioka, Anna Nisyraiou, Antonios Bozas, Maria Vasilopoulou, Konstantina Stavrogianni, Maria Karekla, Marianna Zaharia, Anastasia Iatrou, Christina Karamanidou

**Affiliations:** 1 Institute of Applied Biosciences, Centre for Research and Technology Hellas, Thessaloniki, GRC; 2 ACThealthy Laboratory, Department of Psychology, University of Cyprus, Nicosia, CYP

**Keywords:** acceptance and commitment therapy (act), non-randomized controlled study, pilot study, posttraumatic growth (ptg), psychological distress

## Abstract

Background: Post-traumatic growth (PTG) is characterized by emotional, cognitive, and existential change and is associated with higher spiritual well-being and lower distress. Although Acceptance and Commitment Therapy (ACT) effectively reduces anxiety, depression, trauma symptoms, and fear of cancer recurrence in cancer survivors, PTG has rarely been examined in randomized or controlled trials. This pilot non-randomized controlled study evaluated an ACT-based intervention to promote PTG among Greek cancer survivors and explored its effects on psychological and physiological stress outcomes.

Methodology: A pilot pre-post non-randomised controlled clinical trial design was conducted with 65 participants diagnosed with breast, colorectal, or head and neck cancer, recruited from two hospital oncology clinics. Participants were allocated to either an Intervention Group (IG), receiving a six-week ACT group program, or a Control Group (CG). Measures of PTG, anxiety, depression, traumatic stress, and salivary cortisol were collected at baseline (T1) and post-intervention (T2).

Results: At baseline, groups were comparable across demographic and clinical variables. Following the intervention, the IG showed significant increases in overall PTG and in the Personal Strength factor. Anxiety and cortisol levels significantly decreased in the IG compared with the CG, whereas reductions in depression and traumatic stress did not reach significance after adjusting for baseline scores.

Conclusion: Findings suggest that a brief ACT-based intervention can meaningfully enhance key dimensions of PTG and reduce anxiety among cancer survivors. As a pilot non-randomised controlled study, effect sizes and feasibility outcomes support the development of larger, fully powered trials incorporating long-term follow-up and biological stress markers.

## Introduction

There are nearly 20 million new cancer cases worldwide, and approximately one in five people will develop cancer in their lifetime [1]. Overall, cancer incidence increased by 28-47% between 2010 and 2020, with the sharpest increases seen in low and middle-Sociodemographic Index (SDI) countries, underscoring major global disparities in the cancer burden, while imposing substantial economic and psychosocial costs [1,2].

 Psychological impact, social relationships, and quality of life among cancer survivors

Cancer, as a major life stressor, goes beyond physical symptoms, often leading to considerable psychological distress and the development of mental health disorders [3], with a compromised psychological state being associated with lower quality of life, reduced psychosocial functioning [4], and poorer prognosis [5]. Psychosocial distress is highest at diagnosis and persists in over 30% of survivors up to 10 years post diagnosis, with improvement in some and worsening in others [6]. Depression, anxiety, post-traumatic stress disorder (PTSD), and sleep disturbances are the most prevalent mental health disorders among cancer patients. A recent meta-analysis reported an anxiety prevalence of 24.4%, a depression prevalence of 23.7%, a sleep disorder prevalence of 34.1%, and a PTSD prevalence of 19.9%, with overall rates decreasing over time since diagnosis [7]. Regarding cognitive health, the pooled prevalence of cognitive impairment among breast cancer survivors is 21-34%%, indicating that nearly one in three survivors experience measurable cognitive difficulties following treatment [8].

Beyond the psychological distress and the development of mental health disorders, cancer can deeply affect one’s sense of self, interpersonal relationships, and overall social functioning. The diagnosis and treatment can disrupt core aspects of self-concept, including self-esteem, self-discontent, self-appraisal, and body image, which in turn affect psychological adjustment, treatment outcomes, and social functioning [9]. Physical changes from treatment, such as scarring, hair loss, or altered body shape, often lead to body image dissatisfaction and psychological distress, with fear of these changes sometimes arising even before treatment [10]. Women with breast cancer and patients with head and neck cancer are particularly vulnerable, as they navigate the impact of bodily changes on personal identity and societal expectations [11,12]. Cancer and its treatment can lead to sexual dysfunction, negatively affecting quality of life and intimacy for patients and survivors [13], with reduced sexual satisfaction and activity reported across ages and cancer types. Social relationships are also profoundly impacted, with delays in romantic involvement, lower marriage rates, marital distress, and altered family dynamics, including changes in expressiveness and support [14-16]. Broader social functioning may be further disrupted by unemployment, social isolation, and experiences of criticism or cancer-related loneliness [17,18].

Acceptance and commitment therapy (ACT) as a psychological intervention for cancer survivors

A wide range of interventions has been developed for cancer survivors, but they commonly share key therapeutic components such as an emotionally supportive environment and elements of education, emotional processing, skill building, like enhancing decision-making and coping, stress management, and relaxation training. Psychoeducational interventions provide structured information on disease, treatment, symptom management, and coping strategies, reducing distress, enhancing self-efficacy, and supporting treatment adherence [19]. Relaxation and stress management techniques, including progressive muscle relaxation, guided imagery, and diaphragmatic breathing, lower physiological arousal and stress, improving coping, sleep, pain tolerance, and overall well-being [20]. Couples- and family-based interventions improve communication, support, and shared coping, enhancing relationship satisfaction, psychological well-being, and adherence, particularly in family-centered cultures [21]. 

Cognitive behavioral therapy (CBT) helps patients challenge negative thoughts and develop adaptive coping, reducing anxiety, depression, fatigue, and improving quality of life and adherence [22]. Third wave interventions such as mindfulness promote present-moment awareness and mind-body connection, reducing stress, anxiety, fatigue, and pain [23]. Besides, meaning-centered interventions help patients explore purpose and values, enhancing spiritual well-being, psychological adjustment, and quality of life while reducing distress, particularly with patients with advanced disease [24]. Another third-wave CBT intervention, ACT, is a transdiagnostic, process-based approach that promotes acceptance, mindfulness, cognitive diffusion, and values-based actions, helping patients confront difficult emotions and thoughts with flexibility rather than avoidance or trying to change/eliminate [25]. ACT reduces depression, anxiety, and psychological distress, while improving health-related quality of life (HRQoL) and emotional well-being in cancer patients and survivors. It may also positively impact physical outcomes such as fatigue and inflammation, representing an important addition to psycho-oncological support strategies [26,27].

The concept of post-traumatic growth (PTG)

Several psychotherapeutic interventions, including third-wave therapies such as meaning therapy, mindfulness, and ACT, have shown promise in enhancing PTG in cancer populations [28-30]. According to Tedeschi and Calhoun (2004), PTG refers to the positive psychological changes that emerge from struggling with highly challenging or traumatic life events [31]. It can manifest as a deeper appreciation of life, stronger and more meaningful relationships, greater personal strength, a shift in priorities, and an enriched existential or spiritual outlook. 

PTG represents a profound qualitative shift in adaptation, extending beyond resilience or the ability to withstand stress and surpassing pre-trauma levels of functioning, thereby distinguishing it from related concepts such as sense of coherence, optimism, and hardiness [32]. It comprises five dimensions: greater appreciation of life and a changed sense of priorities; warmer, more intimate relationships with others; a greater sense of personal strength; recognition of new possibilities or paths for one’s life; and spiritual development [33]. It is influenced by personal traits such as extraversion, openness, and optimism, and emerges through coping with intense emotions, followed by deep cognitive processing of the event and the development of new schemas, goals, and meanings. Social support further facilitates growth by helping individuals create coherent narratives and providing perspectives that inform revised cognitive schemas, often leading to greater wisdom, a renewed life narrative, and personal meaning [31]. Although distress may lessen over time, some psychological struggle is essential to initiate and sustain growth.

Aim of the study

PTG involves emotional, cognitive, and existential shifts and is shaped by various psychological and physiological factors. Key outcomes such as spiritual well-being, lower distress, and depression can act as both predictors and results of PTG [34]. Although ACT is effective for anxiety, depression, posttraumatic stress, psychological flexibility, fear of concurrent cancer, and quality of life in cancer survivors, PTG has not been widely assessed in many randomized controlled trials (RCTs) [35]. Building on this rationale, the primary aim of this non-randomized controlled pilot study was to evaluate the efficacy of an ACT-based intervention in promoting PTG among Greek patients with breast, colon, or head and neck cancer. The secondary aim was to examine ACT-based effects on psychological and physiological outcomes, including anxiety, depression, posttraumatic stress impact, and stress biomarkers.

We hypothesize that, compared with the control group, the ACT-based intervention will demonstrate: (i) higher PTG; (ii) lower levels of anxiety, depression, and posttraumatic stress; and (iii) lower salivary cortisol levels, indicating reduced physiological stress.

As a non-randomized controlled pilot study, the results of this trial are intended to evaluate feasibility and generate preliminary evidence to inform the development of targeted, evidence-based interventions aimed at enhancing well-being and resilience in oncology care. These findings will lay the groundwork for future fully powered, randomized studies and support the advancement of more personalized therapeutic strategies.

## Materials and methods

Protocol development

To develop the protocol, a scoping review was conducted to synthesize and map recent evidence on PTG in adult cancer populations. Findings from the scoping review provided the conceptual foundation for the protocol, guiding the selection of intervention components, informing tailoring strategies for the cancer context, and supporting the choice of outcome measures for the pilot trial. Further details are reported elsewhere [36].

Additionally, a systematic review and meta-analysis was conducted to identify psychosocial interventions that may enhance PTG, as measured by the Posttraumatic Growth Inventory (PTGI), among adults with cancer and to evaluate their effectiveness. Further details are reported elsewhere [37].

At the same time, parallel action took place involving hospital clinic directors (July 2024-October 2024). Initial meetings between the research team and the participating hospital teams were held to exchange experiences and agree on the actions required to conduct the intervention. In addition, potential barriers were discussed, including challenges in recruiting eligible participants, cancer types meeting the inclusion criteria, and possible dropouts during the intervention

The intervention protocol was developed based on the aforementioned research and the established ACT framework, which was developed and adapted into Greek by Zachariaand Karekla (2021) [38]. The curriculum was further tailored to the specific needs of our cancer population following consultation meetings among the developers, the project lead (CK), one medical psychologist (AB), one clinical psychologist (AN), one counseling psychologist (MV), and one junior psychologist (KS). Each group of participants attended six weekly psychotherapeutic sessions conducted between March 2025 and November 2025. The study is registered in ClinicalTrials.gov under the identifier NCT07108517 [39].

Study design

This interventional study uses a pre-post measurement design within a pilot non-randomized controlled clinical trial. The pilot design was conducted on a small number of participants enrolled at the start of the study to assess feasibility, refine procedures, and confirm key trial parameters, which is particularly useful when there is uncertainty regarding participants’ compliance with the protocol or other operational aspects of the study [40]. The pre-intervention and post-intervention measurements in both the “Intervention Group” (IG) and “Control Group” (CG) were used to evaluate the impact of a six-week program for cancer patients, based on the ACT protocol. Outcome measures were administered before the intervention (Baseline, Time Point 1, T1) and directly after the intervention (Follow-up-Time Point 2, T2) for both IC and CG. 

Setting and participants

The study was conducted in oncology clinics from one general hospital, AHEPA (American Hellenic Educational Progressive Association) University Hospital, and one oncology hospital, Theagenio Cancer Hospital in Thessaloniki, Greece. Participants were recruited between March 2025 and November 2025.

A convenience sample of 65 participants was collected according to specific selection criteria; all participants were diagnosed with one of three types of cancer: breast cancer, head and neck cancer, or colorectal cancer. The inclusion criteria were: (i) adults over 18 years old, (ii) diagnosis of breast, head and neck, or colorectal cancer, (iii) completion of all planned therapies and hospital admissions, and (iv) time since initial diagnosis over two years prior to recruitment. The exclusion criteria included: (i) being in active psychological or psychiatric treatment, (ii) presence of severe psychopathology (e.g., suicidal ideation or psychotic disorders), (iii) inability to speak or read Greek, (iv) not in active remission, (v) severe cognitive impairment, (vi) inability to provide informed consent, (vii) life expectancy of less than one year, and (viii) diagnosis of any other type of cancer within five years prior to recruitment.

In collaboration with healthcare professionals at the oncology clinics, eligible participants were informed about the study during routine outpatient appointments. Clinicians introduced the study and offered additional details to those who expressed interest, engaging them in focused discussions on potential benefits, risks, and expected outcomes to promote adherence. Patients then received an information sheet describing the study, and those who agreed to participate were enrolled after providing written informed consent. A total of approximately 200 patients were initially approached, of whom 90 received full study information, 70 agreed to participate, and 65 finally participated.

Sample Size Calculation

We conducted a power analysis [41] to detect a small to medium effect (f = 0.20, d ≈ 0.40) with ANCOVA (α = 0.05, power = 0.80) and one covariate. A two-arm trial would require roughly 100-200 participants, depending on the strength of the covariate (baseline PTGI). Due to recruitment limitations, our sample size was 65 (30 intervention; 35 control). This sample provides power to detect only moderate to large effects (minimum detectable effect (MDE) ≈ d = 0.6-0.7). Therefore, this study is presented as a pilot/feasibility trial whose main goals are effect-size estimation, feasibility, and informing sample size calculations for a future definitive trial [42].

Procedure

The 65 participants included in the study were allocated to either the IG or the CG based on their preference for receiving psychological support. The IG completed a structured six-week ACT program. Before the start of the intervention, baseline data were collected from all participants from both groups (Time Point 1).

The ACT program was delivered in groups of 8-10 patients with the same cancer type, facilitated by two therapists trained in ACT. Sessions took place at the facilities of a participating AHEPA University General Hospital. The program consisted of six weekly sessions. After the final session, both the IG and CG completed these follow-up assessments at the corresponding time points (Time Point 2). Additionally, at the conclusion of the intervention, feedback was collected from all participants and the healthcare professionals involved in recruitment, providing insights into the study’s feasibility, implementation, and potential improvements for broader application.

Ethics

This study received ethical approval from three independent committees: the Ethics Committee of the coordinating Research Institute, the Centre for Research and Technology Hellas (Date: October 25, 2023, Registry No. 25/10-1), the Scientific Council of AHEPA (Date: May 27, 2024, Registry No. 255), and the Scientific Council of Theagenio Cancer Hospital in Thessaloniki (Date: April 18, 2024, Registry No. 5589).

In accordance with ethical standards, the intervention was also offered to participants in the control group following the completion of the study. This will not be part of the study, and no data will be collected. The intervention would be provided solely for the purpose of supporting participants’ well-being, in recognition of the ethical obligation to offer potentially beneficial treatments according to the Declaration of Helsinki. Participation was entirely voluntary, and participants were informed of their right to withdraw from the study at any point without any consequences or loss of benefits. All data collection, processing, and dissemination of findings were conducted in full compliance with the European Union's General Data Protection Regulation (GDPR).

The intervention of ACT

To promote and enhance PTG in patients with a cancer experience, the research team implemented ACT, an empirically supported psychotherapeutic approach [43]. The intervention was based on the I-CAN-ACT protocol developed by Zacharia and Karekla [38], which is specifically designed to enhance psychological resilience and well-being while reducing psychological distress and physical pain among women with breast cancer. Using the I-CAN-ACT protocol and its core components, the program sought to improve overall well-being by helping participants align their daily actions with personally meaningful values and by encouraging a perspective of the self as a space that can hold all experiences, including those associated with cancer. Besides, the protocol incorporated key ACT principles such as values-based goal setting, acceptance, cognitive diffusion, self-as-context, committed action, self-compassion, and mindfulness [44,45]. The intervention was adapted to the specific needs of the enrolled groups, following consultation with the developers, and was delivered over a six-week period, consisting of six weekly sessions of 90 minutes each. The structure of the ACT protocol sessions is outlined in Table 1. 

**Table 1:** ACT protocol sessions ACT: Acceptance and Commitment Therapy

Session Number	Name of Session	Description
Session 1	Introduction and Values Exploration	Participants are introduced to ACT concepts and begin to explore their personal values. Exercises include the limitations of experiential avoidance and control and the benefits of actions guided by values.
Session 2	Acceptance and Willingness	Emphasis is given on embracing and accepting difficult emotions, distinguishing pain with suffering and practicing mindfulness.
Session 3	Cognitive Defusion and Self-as-Context	Participants are learning distancing from thoughts and the concept of the observing self that it separates self from thoughts.
Session 4	Values-Based Goals and Committed Actions	Focuses on participants setting their values and aligned goals, while they learn how to navigate barriers that may hinder consistent, values-oriented behavior.
Session 5	Commitment, Self-Compassion and Self-as-Context	Emphasizes the importance of commitment to purposeful action and learning self-compassion techniques to foster resilience.
Session 6	Relapse Prevention and Life Beyond Therapy	Presents prevention strategies for relapse and revisits core ACT principles to support ongoing values-driven living.

Measures

To evaluate the impact of the intervention, a set of validated self-report measures was administered. Demographic information was collected from all participants at baseline. The remaining questionnaires were administered at two time points: at baseline (T1) and again following the completion of the six-week intervention period (T2), regardless of group allocation, IG and CG.

Demographics

Demographic information was collected through a self-administered questionnaire, which included information regarding sex, age, nationality, education level, family and work status, economic situation, and religious beliefs/religiosity. Additionally, information related to the disease was gathered, including cancer stage at diagnosis, type and duration of treatment, and the presence of comorbidities.

PTGI

PTGI measures the PTG after a stressful experience, such as cancer. It comprises 21 items grouped into five factors: Relating to Others, New Possibilities, Personal Strengths, Spiritual Change, and Appreciation of Life [33]. Responses are rated on a five-point Likert scale (0 = I did not experience this change as a result of my illness, to 5 = I experienced this change to a very great degree as a result of my illness). Higher scores indicate greater posttraumatic growth. In the present study, the Greek version of the Inventory (PTGI-Gr), which has been administered to patients with advanced cancer, was used. The PTGI-Gr has demonstrated satisfactory psychometric properties. Cronbach's alpha coefficients for the five factors ranged from .66 to .87. The overall test-retest reliability was satisfactory, with values ranging from .85 to .92 (p < .0005) [46].

Impact of Event Scale - Revised (IES - R)

The IES-R is a self-report scale that assesses current psychological distress for a specific life event [47]; in this case, cancer. It consists of 22 items divided into three sub-scales: intrusion, avoidance, and hyperarousal. Participants are asked to rate their responses based on the past seven days using a four-point scale (0 = not at all, 1 = a little bit, 2 = moderately, 3 = quite a bit, and 4 = extremely) with higher scores indicating higher psychological distress. We used the Greek version of IES-R with satisfactory psychometric properties. The test-retest reliability was satisfactory (p < .0005), while the internal consistency was good (α =0.85) [48].

Hospital Anxiety and Depression Scale (HADS)

The HADS is a self-report questionnaire measuring patients’ level of anxiety and depression. It consists of 14 items, divided equally into two subscales: Seven items measuring anxiety and seven measuring depression. Each item is rated on a four-point Likert scale from 0 to 3, yielding a possible score of 0 to 21 for each subscale [49]. The Greek version of the HADS was utilized, which has demonstrated excellent internal consistency in previous Greek validation studies (Cronbach’s alpha = 0.845) [50].

Biomarkers

Stress biomarkers, such as cortisol levels, were assessed through saliva samples [51] collected by trained medical personnel. All saliva samples were collected in the morning between 09:00 and 10:00 a.m. to minimize variability associated with the circadian rhythm of cortisol secretion. Participants were instructed to refrain from eating, drinking caffeinated beverages, smoking, or brushing their teeth for at least one hour prior to sample collection. Individuals with known endocrine disorders or receiving corticosteroid medication were excluded from the study. Saliva was collected using a centrifuge glass tube and a plastic straw with a “Salivette” collection system (Sarstedt AG & Co. KG, Nümbrecht, Germany). The Salivettes were centrifuged at 1000 × g for two minutes to obtain clear saliva. Cortisol concentrations were quantified using the Cortisol Saliva enzyme-linked immunosorbent assay (ELISA) direct competitive immunoenzymatic colorimetric assay (DiaMetra S.r.l, Spello PG, Italy), following the manufacturer’s instructions [52,53]. Absorbance was read on a Spark® microplate reader (Tecan Group Ltd., Männedorf, Switzerland).

Data analysis

Statistical analyses were performed using IBM SPSS Statistics for Windows, version 27.0 (Released 2019; IBM Corp., Armonk, New York, United States). Descriptive statistics (means, standard deviations, and frequencies) were calculated for all demographic and clinical variables. Internal consistency (Cronbach’s α) was tested separately for the baseline and post-intervention administrations of all measures. To evaluate the effectiveness of the intervention, a series of one-way analyses of covariance (ANCOVAs) was conducted for each psychological outcome. Post-intervention scores on the PTGI total scale, PTGI sub-dimensions (Relating to Others, New Possibilities, Personal Strength, Spiritual Change, Appreciation of Life), IES-R, and HADS, were entered as dependent variables, with group (IG vs. CG) as the fixed factor and the corresponding baseline scores as covariates. Adjusted (estimated marginal) means and standard errors were computed for each outcome. Effect sizes were reported using partial eta squared (η²). Assumptions of ANCOVA, including homogeneity of regression slopes, normality of residuals, linearity between covariates and outcomes, and homoscedasticity, were tested prior to analysis. Outliers were examined using standardized residuals and influence statistics. Missing data were evaluated for randomness. For scales with minimal missingness, mean substitution at the item level was used. All hypothesis tests were two-tailed with a significance level set at α = .05. Biomarker data (salivary cortisol) were analyzed using paired-samples t-tests to examine pre-post differences within each group. These analyses were conducted using GraphPad Prism version 9 (Dotmatics, Boston, Massachusetts, United States). Statistical significance for biomarker analyses was set at p < .05.

## Results

Demographics

A total of 65 participants were included in the analysis (IG: 30; CG: 35). Age distributions were comparable across groups, with IG participants ranging from 34 to 76 years (mean = 56.70, SD = 10.15) and CG participants from 39 to 76 years (mean = 56.06, SD = 8.25). The majority of participants for both groups were female (IG=76.7%, n=23; CG= 88.6%, n=31), married (IG=73.3%,n=22; CG= 77.1%, n=27), and had a high school diploma (IG=40%, n=12; CG= 37.1%, n=13). The majority of participants also identified as full-time employees (IG=46.7%, n=14; CG= 64.3%, n=18), followed by those who were retired (IG=30%,n=9; CG= 21.4%, n=6). Regarding economic status, most of them rated their situation as either average (IG: 43.3%, n=13; CG: 48.6%, n=17) or good (IG: 36.7%, n=11; CG: 40%, n=14), which indicates that the sample primarily reflected middle-income households. Finally, religiosity emerged as a notably consistent characteristic. In both groups, 40% (IG, n=12; CG, n=14) of participants rated religion as “very significant” in their lives (Table 2).

**Table 2:** Sociodemographic data of the participants (N=65)

Variables	Categories	Intervention Group (n=30), n (%)	Control Group (n=35), n (%)
Age	Mean±SD	56.70±10.15	56.06±8.25
Sex	Men	7 (23.3)	4 (11.4)
	Women	23 (76.7)	31 (88.6)
Marital Status	Unmarried with a relationship	3 (10)	4 (11.4)
	Married	22 (73.3)	27 (77.1)
	Single	5 (16.7)	4 (11.4)
Education Level	Compulsory education graduate	3 (10)	3 (8.6)
	High school graduate	12 (40)	13 (37.1)
	Higher education student	4 (13.3)	1 (2.9)
	Higher education graduate	9 (30)	12 (34.3)
	Master's degree	2 (6.7)	6 (17.1)
Work Status	Full-time employment	14 (46.7)	18 (64.3)
	Part-time employment	1 (3.3)	1 (3.6)
	Homemaker	2 (6.7)	1 (3.6)
	Retired	9 (30)	6 (21.4)
	Unemployed	4 (13.3)	2 (7.1)
Economic status	Very good	5 (16.7)	3 (8.6)
	Good	11 (36.7)	14 (40)
	Average	13 (43.3)	17 (48.6)
Significance of religion	Very much	12 (40)	14 (40)
	Enough	7 (23.3)	10 (28.6)
	Moderately	9 (30)	6 (17.1)
	Slightly	1 (3.3)	4 (11.4)
	Not at all	1 (3.3)	1 (2.9)

Clinical characteristics

Across both groups, breast cancer was the predominant diagnosis, though it appeared even more frequently in the CG (85.7%, n=30) than in the IG (70%, n=21), followed by head and neck cancers (IG: 10%, n=3; CG: 8.6%, n=3), and colon cancers (IG: 20%, n=6; CG: 5.7%, n=2) which were less common. Time since diagnosis was similarly distributed, with the majority of participants in both groups falling within the two to three-year range post diagnosis (IG: 41.3%,n=12; CG: 40%, n=14), followed by the four to five-year range (IG: 27.5%, n=9; CG: 22.8%, n=8). Notably, the IG included a higher proportion of long-term survivors (>10 years: 17%, n=4) compared to the CG (8.6%, n=3).

Regarding the types of treatment, surgery was mentioned by the vast majority of participants in both groups (IG: 90%, n=27; CG: 91.4%, n=32). Hormone therapy and radiation therapy were also widely reported, with a higher prevalence in the CG (57.1%, n=20 and 74.3%, n=26, respectively) than in the IG (46.7%, n=14 and 66.7%, n=20, respectively). Chemotherapy and immunotherapy were reported almost similarly across groups, with chemotherapy administered to 73.3% (n=22) of the IG and 74.3% (n=26) of the CG, while immunotherapy was reported by 20% of participants in both groups (IG: n=6 and CG: n=7) (Table 3).

**Table 3:** Disease-related characteristics of the participants distributed according to the intervention and control groups

Variables	Categories	Intervention Group (n=30), n (%)	Control Group (n=35), n (%)
Cancer diagnosis	Breast cancer	21 (70)	30 (85.7)
	Head-Neck cancer:	3 (10)	3 (8.6)
	Colon cancer	6 (20)	2 (5.7)
Time since diagnosis	2-3 years	12 (41.3)	14 (40)
	4-5 years	9 (27.5)	8 (22.8)
	6-10 years	5 (17.2)	10 (28.6)
	>10 years	4 (17)	3 (8.6)
Treatment	Surgery	27 (90)	32 (91.4)
	Hormone therapy	14 (46.7)	20 (57.1)
	Radiation therapy	20 (66.7)	26 (74.3)
	Immunotherapy	6 (20)	7 (20)
	Chemotherapy	22 (73.3)	26 (74.3)
Comorbidities	Rheumatic disease	7 (23.3)	1 (2.9)
	Cardiovascular disease	3 (10)	1 (2.9)
	Respiratory disease	1 (3.3)	3 (8.6)
	Diabetes	2 (6.7)	1 (2.9)

Outcome measures

Internal consistency was tested separately for the baseline and post-intervention administrations of all measures. Across all measures (PTGI, HADS, IES) and at both time points (T1 and T2), Cronbach’s α values ranged from .75 to .95, indicating acceptable to excellent reliability and supporting the consistent use of these instruments in the present study.

Post-traumatic Growth (PTGI)

A one-way ANCOVA was conducted to examine the effect of the intervention on post-intervention PTGI total scores, controlling for baseline PTGI ‘’total scores’’ (n=65). After adjusting for PTGI ‘’total score’’ T1, there was a significant effect of Intervention on PTGI ‘’total score’’ on T2, F(1, 62) = 4.36, p = .041, partial η² = .066. Participants in the intervention group had significantly higher adjusted post-intervention PTGI scores (M = 76.65, SE = 3.31) than controls (M = 67.24, SE = 3.06). The covariate (baseline PTGI) was significant, F(1, 62) = 28.11, p < .001, partial η² = .312. Exploring the factors of the PTGI, five further one-way ANCOVAs were conducted. A statistically significant effect of the intervention was observed on the PTGI ‘’Personal Strength’’ factor at post-intervention (T2), after controlling for baseline scores (T1), F(1, 62) = 11.08, p = .001, partial η² = .152. Adjusted means indicated that the intervention group (M = 16.76, SE = .64) scored significantly higher than controls (M = 13.87, SE = .59). The covariate was also significant, F(1, 62) = 16.54, p < .001. Controlling for baseline scores on the ‘’Relating to Others’’ factor (T1), the effect of the intervention on post-intervention scores (T2) was not statistically significant, F(1, 62) = 3.11, p = .083, partial η² = .048. Adjusted means indicated a non-significant trend favoring the intervention group (M = 24.79, SE = 1.19) relative to the control group (M = 21.93, SE = 1.10). The covariate was significant, F(1, 62) = 37.51, p < .001. Besides, the effect of the intervention on post-intervention scores (T2) of the ‘’New Possibilities’’ factor after controlling for baseline scores (T1) was not statistically significant, F(1, 62) = 2.94, p = .091, partial η² = .045. Adjusted means indicated a non-significant trend favoring the intervention group M = 16.64 (SE = .92), and control M = 14.48 (SE = .85). The covariate was significant, F(1, 62) = 29.17, p < .001. Moreover, after adjusting for baseline scores, intervention did not significantly affect PTGI factor ‘’Appreciation of Life’’ (T2), F(1, 62) = 0.96, p = .331, partial η² = .015. The covariate was significant, F(1, 62) = 21.74, p < .001. Lastly, the effect of intervention on PTGI factor ‘’Spiritual Change’’ (T2) was not significant, F(1, 62) = 1.61, p = .210, partial η² = .025. Baseline scores were strongly predictive, F(1, 62) = 61.74, p < .001. Means and standard deviations at baseline (T1) and follow-up (T2) are presented in Table 4. 

**Table 4:** Means and standard deviations for baseline and post-intervention outcomes by group Note. T1 = Baseline; T2 = post-intervention. PTGI: Post-Traumatic Growth Inventory; HADS: Hospital Anxiety Depression Scale; IES-R: Impact of Events Scale-Revised

Measure	Intervention, mean	Intervention, SD	N	Control, mean	Control, SD	N
PTGI Total (T1)	65.73	24.95	30	66.97	21.10	35
PTGI Total (T2)	76.30	17.54	30	67.54	24.61	35
Relating to Others (T1)	21.73	9.27	30	22.34	8.49	35
Relating to Others (T2)	24.60	6.72	30	22.09	9.27	35
New Possibilities (T1)	14.10	6.68	30	13.31	6.00	35
New Possibilities (T2)	16.87	5.69	30	14.29	6.37	35
Personal Strength (T1)	13.47	5.04	30	14.34	3.86	35
Personal Strength (T2)	16.57	2.28	30	14.03	4.84	35
Spiritual Change (T1)	5.47	3.55	30	5.77	2.65	35
Spiritual Change (T2)	6.37	2.91	30	5.91	2.90	35
Appreciation of Life (T1)	10.97	4.20	30	11.20	4.45	35
Appreciation of Life (T2)	11.90	3.24	30	11.23	3.96	35
HADS Anxiety (T1)	7.32	4.05	30	6.56	4.03	35
HADS Anxiety (T2)	6.14	3.33	30	7.15	4.13	35
HADS Depression (T1)	4.76	2.26	30	4.48	3.75	35
HADS Depression (T2)	4.32	3.20	30	4.67	3.83	35
IES-R Total (T1)	33.13	16.91	30	38.00	21.18	35
IES-R Total (T2)	29.70	16.99	30	38.34	22.28	35

Anxiety (HADS-A)

A one-way ANCOVA was conducted to examine the effect of the intervention on post-intervention anxiety (HADS-A total score, n=65). Controlling for baseline anxiety, the effect of the intervention on post-intervention HADS Anxiety total score T2 was significant, F(1, 46) = 3.78, p = .050, partial η² = .076. Adjusted means indicated lower anxiety in the intervention group (M = 5.86, SE = .58) compared to controls (M = 7.38, SE = .52). The covariate remained significant, F(1, 46) = 46.19, p < .001. Means and standard deviations at baseline (T1) and follow-up (T2) are presented in Table 4.

Depression (HADS-D)

Another one-way ANCOVA was conducted to examine the effect of the intervention on post-intervention depression (HADS-B total score, n=65). The ANCOVA revealed no significant effect of the intervention on HADS Depression total score T2, F(1, 45) = 0.43, p = .515, partial η² = .009, whereas baseline depression was a strong predictor, F(1, 45) = 68.31, p < .001. Means and standard deviations at baseline (T1) and follow-up (T2) are presented in Table 4.

IES-R

A final ANCOVA was conducted to examine the effect of the intervention on post-intervention distress caused by a traumatic event (IES-R total score, n = 65). After controlling for baseline IES total score (T1), the intervention effect on IES total score at T2 was not significant, F(1, 62) = 1.93, p = .170, partial η² = .030. Baseline IES was significant, F(1, 62) = 39.65, p < .001. Means and standard deviations at baseline (T1) and follow-up (T2) are presented in Table 4. 

Biomarkers

Regarding the primary outcome ascertainment rate, the number of participants at both T1 and T2 was n = 65, corresponding to 100%. Adherence was defined as completing at least four out of six sessions. The number of participants who met the adherence-to-intervention criterion was 29, representing > 60% of participants, who completed four or more sessions. The adherence rate for the cortisol measure was approximately 28% (n=18) at both T1 and T2.

**Figure 1:**
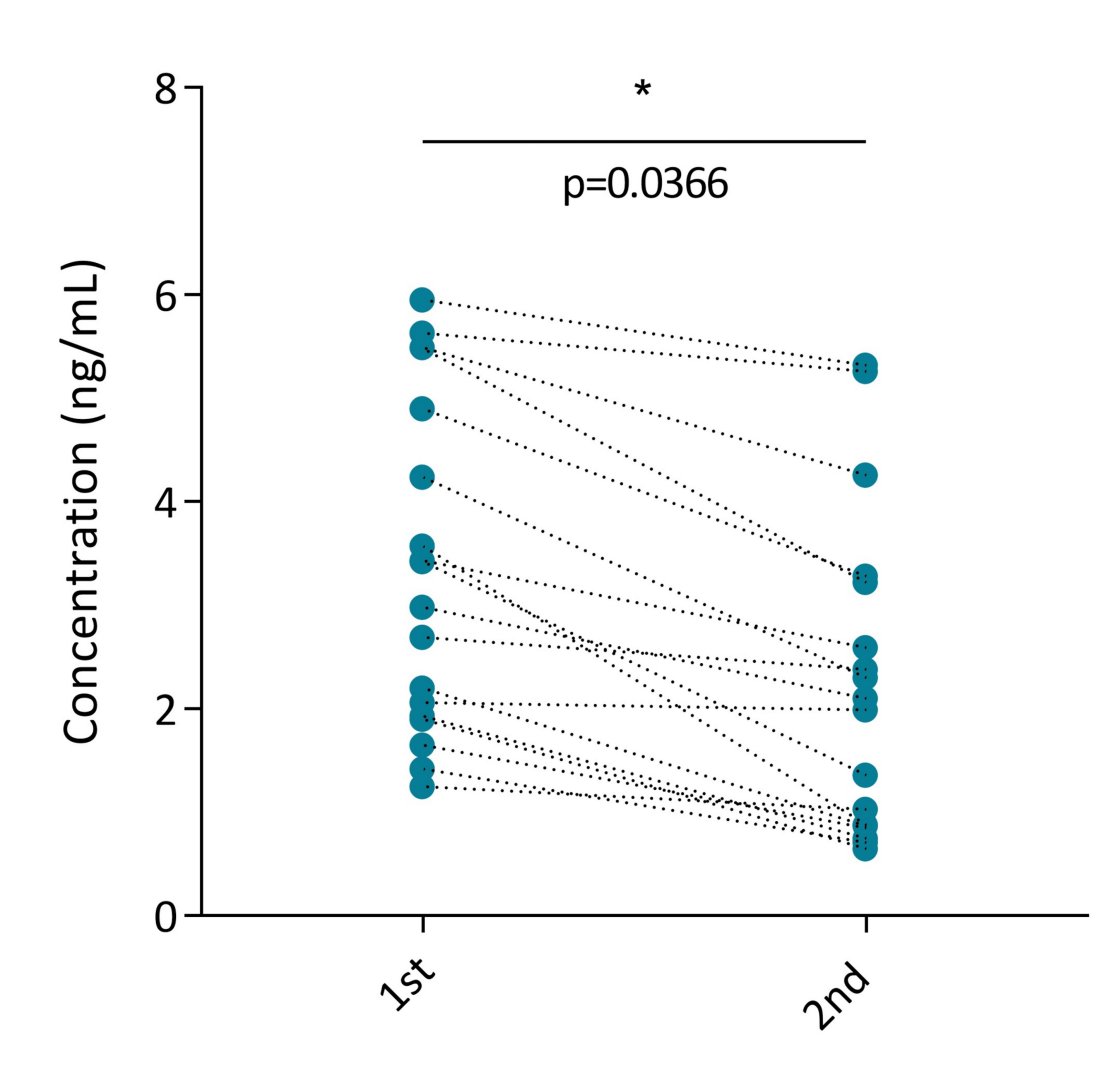
Salivary cortisol concentration in T1 and T2 Salivary cortisol concentrations at two time points. Each point represents an individual participant (n=18), with dotted lines connecting paired before-after values. A significant reduction in cortisol levels was observed at the second time point compared with the first one (paired t-test, p = 0.0366; fold change = 0.66).

In the subset of participants with measurements at T1 and T2 (n=18), a paired before-and-after analysis was performed to evaluate changes in salivary cortisol levels. As shown in Figure 1, cortisol levels were significantly reduced at the second timepoint (p=0.0366; fold change: 0.66). 

## Discussion

The aim of the present study was primarily to evaluate the efficacy of an ACT-based intervention in promoting PTG among Greek patients with breast, colon, or head and neck cancer, and secondarily to examine the intervention’s effects on psychological and physiological outcomes, including anxiety, depression, the perceived impact of the cancer experience/post-traumatic stress, and stress biomarkers. This is the first study in Greece to specifically measure PTG as an outcome of ACT. According to descriptives, participants with comparable mean ages were predominantly female in sex, married, and of middle-income status. Breast cancer was the most common diagnosis, followed by head and neck and colon cancers, with most participants having two to five years post-diagnosis experience, and the IG included a higher proportion of long-term survivors (>10 years). Surgery was the most frequent treatment, while hormone therapy, radiation, chemotherapy, and immunotherapy were also reported across groups. Comorbidities were generally low, though rheumatic and cardiovascular conditions were more common in the IG, and respiratory diseasewas slightly higher in the CG.

According to the main outcome findings, the IG and CG, at baseline, were largely comparable across measures. Following the protocol, participants in the IG demonstrated significant improvements in PTG, particularly in overall PTGI and the Personal Strength factor of PTGI, indicating enhanced global growth and perceived resilience. Trends favoring the intervention were observed for the Relating to Others and New Possibilities factors, though these were not statistically significant. For emotional distress, the intervention group showed reductions in anxiety and depression, with significant between-group differences observed only for anxiety, while cortisol levels also decreased following the intervention, indicating reduced physiological stress. Traumatic stress symptoms (IES) decreased in the intervention group and slightly increased in the control group, but these differences were not significant after controlling for baseline scores. Overall, the findings indicate a targeted and meaningful impact on core dimensions of posttraumatic growth, especially those related to inner resilience and strength, and reduction of anxiety.

Across the literature, the interventions showing moderate to strong effects on PTG include CBT, mindfulness-based approaches, educational programs, and peer-support or health-coaching models [37]. To date, very few studies have investigated PTG via PTGI following ACT. Notably, only one quasi-experimental controlled study (n=20) has specifically measured PTG using the PTGI and reported significant differences between groups among breast cancer patients [54]. Like our study, these findings provide preliminary evidence for the potential benefits of ACT in enhancing post-traumatic growth.

Regarding depression, anxiety, and post-traumatic stress outcomes, similar findings have been reported in previous preliminary studies, reviews, and meta-analyses of RCTs [25,27] and non-RCTs [55-59]. Specifically, across systematic reviews and meta-analyses, the effects of ACT on psychological outcomes in cancer populations show both consistency and variability. Regarding anxiety, depression, and psychological distress, a recent review in advanced cancer reported significant reductions in all three outcomes [27], which aligns with the findings of a meta-analysis in breast cancer survivors, showing moderate to large decreases in anxiety, depression, and stress, along with an enhancement in hope [59]. Similarly, a more recent systematic review and meta-analysis that included RCTs, non-RCTs, and single-arm designs confirmed these benefits, demonstrating consistent reductions in anxiety, depression, psychological distress, cancer-related trauma symptoms, and fear of cancer reoccurrence, while also promoting self-compassion and emotional approach coping among cancer patients and survivors [25].

In line with these findings, our study also observed a reduction in anxiety among participants who received the ACT-based intervention, supporting its beneficial role in alleviating emotional distress in this population. Moreover, findings on dimensions of quality of life were also generally supportive. There are significant and consistent improvements in HRQoL and fatigue among advanced cancer survivors [27] and cancer survivors. In contrast, evidence regarding psychological flexibility, a core therapeutic mechanism of ACT, was mixed across reviews. In two systematic reviews, psychological flexibility, fear of cancer reoccurrence and pain were not significantly improved, with findings reported as inconclusive [59,25]. However, in a broader meta-analysis, significant improvements in psychological flexibility were demonstrated [25], suggesting that effects may vary by study population or study design.

Although no clinical trials of ACT in cancer survivors have yet reported reductions in cortisol, other psychosocial and behavioral interventions have shown promising effects on neuroendocrine stress markers. Specifically, a meta‑analysis of psychosocial interventions in breast cancer patients demonstrated overall large effect sizes for reductions in cortisol levels, whether measured in blood or saliva [60]. Among the most effective psychological interventions were Body-Mind-Spirit (BMS), a meditation-based program called Cognitively-Based Compassion Training (CBCT), Cognitive-Behavioral Stress Management (CBSM), and Mindfulness-Based Stress Reduction (MBSR). These findings suggest that interventions targeting stress regulation might be able to modulate hypothalamic-pituitary-adrenal (HPA) axis activity in cancer survivors, which is in line with the results of the current study. Nevertheless, the evidence most strongly supports mind-body interventions for preserving diurnal cortisol rhythm in cancer survivors, particularly when measured using multi-day sampling protocols assessing slope rather than total output alone [61]. Given the central role of psychological flexibility in ACT, future trials should include cortisol or other biomarkers to test whether ACT directly affects physiological stress regulation.

Limitations and future directions

This study has several limitations. As a pilot non-randomized controlled trial, the small sample size limits the generalizability of our findings. External validity is constrained because the sample may not represent the broader cancer survivor population, and our results may not fully apply to more diverse clinical or community settings. Moreover, pilot designs often lack the statistical power to detect smaller effects, and the absence of randomization increases the risk of baseline imbalances, self-selection biases, and unmeasured confounding that may influence outcomes [62]. Preference-based allocation was adopted in the current study for pragmatic reasons, reflecting real-world conditions and facilitating participation in a psychosocial intervention; however, it also introduces an inherent risk of selection bias, as improvements in posttraumatic growth and anxiety may partially reflect pre-existing psychological differences rather than the intervention effect alone. To partially address these key limitations, baseline adjustment using ANCOVA was applied to reduce baseline confounding and mitigate selection bias associated with non-randomized group allocation [63]. Despite the use of appropriate statistical adjustment, causal inferences should be interpreted with caution.

As a pilot study, our findings provide only preliminary indications of feasibility and potential efficacy and therefore require confirmation in larger, rigorously designed studies. Future research should employ randomized controlled designs or advanced causal inference approaches, such as propensity score-based methods, together with more comprehensive baseline psychosocial assessments, to support stronger causal conclusions

## Conclusions

The present study provides preliminary evidence that an ACT-based intervention can enhance PTG, particularly in global growth and personal strength, while also reducing anxiety among Greek cancer survivors. The findings demonstrate the effectiveness of ACT in improving psychological outcomes, quality of life, and, potentially, stress regulation. Future research should focus on larger RCTs to confirm these effects, include long-term follow-up assessments, and incorporate additional biological outcomes (e.g., inflammatory markers) or study designs such as multi-day sampling protocols for cortisol, to better understand the mechanisms underlying ACT. Additionally, examining ACT’s impact across diverse cancer populations and stages, and exploring its effects on dimensions such as fear of recurrence and resilience, will be essential for optimizing and personalizing interventions for survivors.
